# SIRT3 alleviates painful diabetic neuropathy by mediating the FoxO3a‐PINK1‐Parkin signaling pathway to activate mitophagy

**DOI:** 10.1111/cns.14703

**Published:** 2024-04-04

**Authors:** Jing Yang, Zhuoying Yu, Ye Jiang, Zixian Zhang, Yue Tian, Jie Cai, Min Wei, Yanhan Lyu, Dongsheng Yang, Shixiong Shen, Guo‐Gang Xing, Min Li

**Affiliations:** ^1^ Department of Anesthesiology Peking University Third Hospital Beijing China; ^2^ Neuroscience Research Institute, Peking University Beijing China; ^3^ Department of Neurobiology, School of Basic Medical Sciences Peking University Health Science Center Beijing China; ^4^ Key Laboratory for Neuroscience Ministry of Education of China and National Health Commission of China Beijing China

**Keywords:** dorsal root ganglion, mitochondria, mitophagy, painful diabetic neuropathy, SIRT3

## Abstract

**Introduction:**

Painful diabetic neuropathy (PDN) is a common complication of diabetes. Previous studies have implicated that mitochondrial dysfunction plays a role in the development of PDN, but its pathogenesis and mechanism have not been fully investigated.

**Methods:**

In this study, we used high‐fat diet/low‐dose streptozotocin‐induced rats as a model of type 2 diabetes mellitus. Behavioral testing, whole‐cell patch‐clamp recordings of dorsal root ganglion (DRG) neurons, and complex sensory nerve conduction velocity studies were used to assess peripheral neuropathy. Mitochondrial membrane potential (MMP), ATP, tissue reactive oxygen species, and transmission electron microscopy were used to evaluate the function and morphology of mitochondria in DRG. Real‐time PCR, western blot, and immunofluorescence were performed to investigate the mechanism.

**Results:**

We found that damaged mitochondria were accumulated and mitophagy was inhibited in PDN rats. The expression of sirtuin 3 (SIRT3), which is an NAD^+^‐dependent deacetylase in mitochondria, was inhibited. Overexpression of SIRT3 in DRG neurons by intrathecally administered LV‐SIRT3 lentivirus ameliorated neurological and mitochondrial dysfunctions. This was evidenced by the reversal of allodynia and nociceptor hyperexcitability, as well as the restoration of MMP and ATP levels. Overexpression of SIRT3 restored the inhibited mitophagy by activating the FoxO3a‐PINK1‐Parkin signaling pathway. The effects of SIRT3 overexpression, including the reversal of allodynia and nociceptor hyperexcitability, the improvement of impaired mitochondria and mitophagy, and the restoration of PINK1 and Parkin expression, were counteracted when FoxO3a siRNA was intrathecally injected.

**Conclusion:**

These results showed that SIRT3 overexpression ameliorates PDN via activation of FoxO3a‐PINK1‐Parkin‐mediated mitophagy, suggesting that SIRT3 may become an encouraging therapeutic strategy for PDN.

## Graphical abstract

Painful diabetic neuropathy in the dorsal root ganglion (DRG) induces mitochondrial impairment and sirtuin 3 deficiency, leading to inhibited mitophagy through the FoxO3a‐PINK1‐Parkin pathway, exacerbating allodynia.

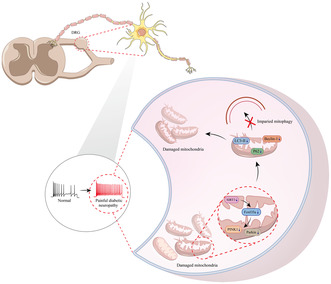

## 1 INTRODUCTION

The prevalence of type 2 diabetes mellitus (T2DM) is on the rise worldwide. Neuropathy is one of the most common long‐term complications of diabetes and approximately 30%–50% of patients with diabetic neuropathy develop neuropathic pain (NP).^1^ The symptoms of painful diabetic neuropathy (PDN) include neuropathic pain and nerve fiber damage. The etiology of NP resulting from diabetes is multifactorial, including metabolism disorder, insulin signaling disturbance, inflammatory, and immune responses.^2^ More importantly, emerging evidence has shown that mitochondrial dysfunction plays a critical role in PDN.^3^, ^4^

Mitophagy is an important way among mitochondrial clearance pathways. Together with mitochondrial fission and fusion, and mitochondrial biogenesis, mitophagy is one of the most important steps to maintain the quality of mitochondria.^5^ Inhibited mitophagy causes the accumulation of dysfunctional mitochondria, provoking diverse pathological processes of diseases. Recent studies have suggested that the impairment of mitophagy induces mitochondrial dysfunction, thereby accelerating the development of diabetic cardiomyopathy and diabetic nephropathy.^6^, ^7^ However, the impact of inhibited mitophagy on the development of PDN remains inadequately explored.

Sirtuin 3 (SIRT3) is an NAD^+^‐dependent deacetylase in the mitochondria with an extensive ability to regulate mitochondrial morphology and function, such as mitochondrial oxidative stress, mitochondrial homeostasis, and mitochondrial biogenesis.^8^ A previous study showed SIRT3 inhibits oxidative stress, therefore alleviating pain in T2DM rats.^9^ Recent evidence indicated that SIRT3 could potentially regulate mitophagy in certain diseases.^10^, ^11^ Upregulation of SIRT3‐mediated mitophagy in cardiomyocytes plays a protective role against myocardial ischemia‐induced heart failure.^12^ Similarly, targeting SIRT3 to activate mitophagy has a therapeutic effect on attenuating advanced glycation end products (AGEs)‐associated senile osteoporosis.^13^ The impact of SIRT3 on mitophagy in PDN remains inadequately explored.

Forkhead box transcription factor 3a (FoxO3a) is a transcription factor that regulates multiple cellular functions, such as cell proliferation, invasive migration, and cell death.^14^ Previous studies have demonstrated that the expression of FoxO3a can be modulated through various mechanisms, including the modification of FoxO3a deacetylation,^15^ phosphorylation,^9^ ubiquitination,^9^ nuclear translocation,^16^ and FoxO3a‐dependent gene expression.^17^

Based on the research findings mentioned previously, we propose the hypothesis that the inhibited mitophagy takes place during the development of PDN, and SIRT3 may play a role in the process of mitophagy through FoxO3a‐mediated mechanisms.

## 2 MATERIALS AND METHODS

### 2.1 Animals and treatment

Healthy male SD rats (6–8 weeks, 160–200 g) were purchased from the Department of Laboratory Animal Research Center, Peking University, and kept in conventional laboratory settings. Two rats were housed in each cage, and they were given free access to food and water for a week while becoming acclimated in a controlled environment with 24 ± 1°C, 60% humidity, and a 12:12 h light–dark cycle. All animal experiments were reviewed and approved by the Peking University Animal Care and Use Committee (no. LA2020057). There were two diets available: a regular chow diet and a high‐fat diet (HFD; Research Diets, catalog D12451). On a caloric basis, the chow diet contained 11.5% fat, 20.8% protein, and 67.7% carbohydrate, while the HFD contained 45% fat, 20% protein, and 35% carbohydrate. After 7‐day acclimating to the animal facility, the rats were randomly divided into 2 groups. The hyperglycemic state was induced by a single intraperitoneal injection of streptozotocin (STZ) (Sigma‐Aldrich Co, St Louis, MO) at 30 mg/kg after 8 weeks on a HFD. After 5 days of streptozotocin injection, rats with fasting blood‐glucose values ≥11.1 mmol/L were considered T2DM rats (Figure S1A,B). On day 7 after STZ injection, an intraperitoneal glucose tolerance test (IPGTT) was conducted after 12 h fasting. 50% glucose solution (2 g/kg) was administered intraperitoneally, and blood glucose levels were measured before and after injection at 30, 60, 90, and 120 min, respectively (Figure S1C). On the 14th day after STZ injection, an insulin tolerance test (ITT) was performed after 12 h fasting. Moreover, regular insulin (Novo Nordisk R, Denmark) was injected intraperitoneally at 0.5 U/kg, and blood glucose levels were measured before and after injection at 30, 60, 90, and 120 min, respectively (Figure S1D).

### 2.2 Lentiviral constructs

Produce a recombinant lentivirus expressing SIRT3 fused with EGFP (referred to as LV‐SIRT3), using a vector containing pCLenti‐mCMV‐EGFP obtained from OBiO Technology (Shanghai) Corp., Ltd. Validation of the transfection efficiency of lentivirus infection was performed on primary cultured dorsal root ganglion (DRG) neurons. Cultured DRG neurons on day 3 were exposed to the virus for 48 h at a multiplicity of infection (MOI). About 48 h after viral infection, the transfection efficiency of lentivirus was routinely achieved as confirmed by observations using fluorescence microscopy. For the in vivo studies, lentivirus, including LV‐SIRT3 and its control LV‐GFP, was administered intrathecally at the dose of 2 × 10^8^ IFU/10 μL (in a 25‐μL volume of solution), respectively.

### 2.3 siRNA preparation and screening

Targeted siRNA was applied to knock down the expression of FoxO3a. siRNA targeting FoxO3a (5′‐GCACCAUGAAUCUGAACGATT‐3′) and scrambled siRNA (5′‐UCGUUCAGAUUCAUGGUGCTT‐3′) were purchased from GenePharma Company (Suzhou, China). In this study, we administered a 10‐μL volume of FoxO3a small interfering RNA (FoxO3a siRNA) via intrathecal injection into the DRG, using a 2‐μg dosage. The efficiency of transfection was validated by western blot.

### 2.4 Behavioral tests

Pain behavior tests were assessed at the start of the trial (BL), at days 7, 14, 21, 28, and 35 following the injection of STZ, and at days 13, 14, 21, 23, 25, 27, and 29 following the intrathecal injection of lentivirus or siRNA. All behavioral experiments were performed blinded to the treatment groups.

#### 2.4.1 Assessment of mechanical allodynia

The 50% paw withdrawal threshold (PWT), as reported in an earlier finding,^18^ was used to measure mechanical allodynia. The Up‐and‐Down approach was used to determine the 50% PWT in response to a set of von Frey filaments. Rats were put in an inverted clear plastic cage with a metal mesh floor. Eight von Frey strands with about similar incremental logarithmic (0.224) bending forces were selected (0.41, 0.70, 1.20, 2.00, 3.63, 5.50, 8.50, and 15.10 g). A 2.0 g von Frey force applied perpendicularly to the left hind paw's plantar surface for 2–3 s at the beginning of each trial. The shrinking of the hind paw was recorded as a positive reaction.

#### 2.4.2 Assessment of thermal hyperalgesia

According to an earlier study, the paw withdrawal latency (PWL) was tested.^19^ Rats were given 30 min to adjust in acrylic cages on a transparent glass plate. The light source was directed vertically onto a specific area of the skin on the hind limb. The thermal stimulation was interrupted when the rat lifted its hind paw, and the time was recorded as the latency period of the heat‐retracted reflex. Each rat had its three PWL measurements, and the results of each test were averaged.

### 2.5 Implantation of intrathecal catheter

The implantation of an intrathecal cannula was carried out according to a previous procedure^20^ after anesthesia with pentobarbital (0.03 g/kg, i.p.). Briefly, a PE‐10 polyethylene catheter was implanted between the L5 and L6 vertebrae and the operation was considered successful if the rat exhibited a tail‐flick response. Through the implanted catheter, vehicles were delivered in a 10‐μL vote lumen of the solution, followed by a 10‐μL vote lumen of the vehicle for flushing. Each injection lasted at least 5 min, and the needle was left in place for 2 min before being removed.

### 2.6 RNA extraction and real‐time PCR

DRG was treated with a reagent to extract total RNA (Life Technologies). Following the manufacturer's instructions, reverse transcription was performed with Moloney murine leukemia virus reverse transcriptase (Promega) and oligo deoxythymidine (oligo‐dT) primers. PCR was carried out using an ABI 7500 Fast Real‐Time PCR Detection System and PCR Master Mix (Promega) (Applied Biosystems). The PCR primers were as follows: *Sirt3*: 5′‐TACTTCCTTCGGCTGCTTCA‐3′ (forward) and 5′‐AAGGCGAAATCAGCCACA‐3′ (reverse); β‐actin: 5′‐AGCCATGTACGTAGCCATCC‐3′ (forward) and 5′‐GCCATCTCTTGCTCGAAGTC‐3′ (reverse). The relative expression of *Sirt3* was normalized to β‐actin.

### 2.7 Mitochondrial isolation

Tissue mitochondrial extraction kits (C3606, Beyotime) were used for mitochondrial isolation of DRG according to the manufacturer's instructions. The mitochondria and other cytoplasm were isolated through differential centrifugation.

### 2.8 Western blot

DRG and mitochondrial protein were homogenized in radioimmunoprecipitation assay (RIPA) buffer containing phosphatase and protease inhibitor cocktails (Sigma). The experimental protocol was performed in accordance with a previous study.^19^ Antibodies against SIRT3 (ab246522; Abcam), FoxO3a (2497#; Cell Signaling Technology), PINK1 (23274‐1‐AP; Proteintech), Parkin (2132#; Cell Signaling Technology), LC3B (ab48394; Abcam), Beclin‐1 (3495#; Cell Signaling Technology), and P62 (5114#; Cell Signaling Technology) were purchased. GAPDH antibody (2118#; Cell Signaling Technology) and TOM20 (ab186735; Abcam) were used as an internal control.

### 2.9 Primary culture and acute dissociation of DRG neurons

According to the method described in a previous study,^21^ rats (2 weeks old) were used to separate dissociated DRG neurons, which were then plated in a media contained Neurobasal media plus B27 supplement, 0.5 mM L‐glutamic (Sigma‐Aldrich), penicillin (100 U/mL), and streptomycin (100 mg/mL). 3 days after plating, when more than 95% of the cells in the cultures were neurons, they were used for studies.

Acute dissociation of DRG neurons was performed as described in a previous report.^20^ After neurons were isolated from the L4 and L5 DRGs of adult rats and were digested, the dissociated cells were used for patch‐clamp recording within 3 to 8 h.

### 2.10 Transmission electron microscopy (TEM)

DRG was fixed in buffer with 2.5% glutaraldehyde and 0.1 M sodium cacodylate for 24 h. And then, the DRG was embedded in Epon resin as previously described.^22^ Subsequently, 70‐nm sections were obtained and stained with uranyl acetate and lead citrate. The ultrastructure of DRG mitochondria was observed under the Japanese JEM‐1400 transmission electron microscope. Referring to the methods of the previous study,^3^ the sections were first imaged at a low magnification (×500). Once medium–small diameter neurons were identified (<40 μm), images were acquired at ×10,000. Statistical analysis of the number and structure of mitochondria were performed by randomly selecting 12–15 fields from three rats per group. The boundary for each mitochondrion was manually marked using the free‐hand selection tool, and the area and perimeter for each mitochondrion were measured. According to the previous study,^23^ subcellular entities characterized by electron‐dense contents and ensconced within a dual enclosing membrane were discerned as akin to autophagosomes. Among these entities, those encompassing distinguishable mitochondria, as evidenced by the presence of paired membranes and cristae, were recognized as resembling putative mitophagosomes. For the nerve fiber, an average of 11–20 microscopic fields from three rats were analyzed to cover the entire cross‐section of the nerve. The unmyelinated nerve fiber images were acquired at ×10,000 and the myelinated nerve fiber images were acquired at ×1000.

### 2.11 Multi‐label immunofluorescence (mIF)

We used the Opal multi‐label immunofluorescence staining technique. Five‐micrometer formalin‐fixed paraffin‐embedded (FFPE) DRG sections of the rat model were stained. All experiments were detected by immunohistochemistry using a rabbit polyclonal antibody against SIRT3 (ab189860; Abcam), a monoclonal rabbit antibody against FoxO3a (2497#; Cell Signaling Technology), a rabbit polyclonal antibody against PINK1 (23274‐1‐AP; Proteintech), a rabbit polyclonal antibody against Parkin (2132#; Cell Signaling Technology), a rabbit polyclonal antibody COX IV (11242‐1‐AP; Proteintech), a rabbit polyclonal antibody against LC3B (ab48394; Abcam), and a rabbit polyclonal antibody against NeuN (26975‐1‐AP; Proteintech). Whole slide scans were performed using the ×20 objective lens. Acquired images were analyzed by inForm tissue finder software (Akoya bioscience). For the mitophagy immunofluorescence, visualization of fluorescence signal was performed by confocal microscopy at excitation wavelengths of 488 nm (green), 543 nm (red), and 405 nm (blue), and we initially measured under a 64× magnification, selected neurons with a diameter less than 40 μm, and counted the colocalization points.

### 2.12 Mitochondrial membrane potential (MMP)

Mitochondrial membrane potential was measured using JC‐1 (C2003S, Beyotime, China) fluorescence mitochondrial imaging. Purified DRG mitochondrial tissues were mixed with JC‐1 solution and then directly time‐scanned with a fluorescence zymography instrument at an excitation wavelength of 485 nm and an emission wavelength of 590 nm.

### 2.13 Tissue adenosine triphosphate (ATP) detection

ATP levels of DRG were determined using an Enhanced ATP Assay Kit (S0027, Beyotime). The fresh DRG tissue was homogenized in a lysis solution, followed by centrifugation at 12,000 *g* for 5 min at 4°C to collect the supernatant. A reagent was dissolved on an ice bath for later use, and an ATP standard curve was prepared by adding ATP standard solution to the ATP detection solution. ATP detection working solution was then added to the detection wells or tubes and incubated for 5 min to reduce background ATP levels. Subsequently, samples or standard solutions were added and mixed, followed by measurement of the values using a luminometer to calculate ATP concentration based on the standard curve.

### 2.14 Tissue reactive oxygen species (ROS) detection

The DRG tissue was processed on ice, promptly homogenized, lysed, and then centrifuged at 12,000 *g* for 10 min to collect the supernatant. Dichlorodihydrofluorescein diacetate (mak142, Sigma‐Aldrich) was diluted to a concentration of 1:1000 and subsequently was incubated at 37°C for 20 min. The fluorescence intensity was measured with an excitation wavelength of 488 nm and an emission wavelength of 525 nm.

### 2.15 Whole‐cell membrane clamp recording

Whole‐cell patch‐clamp recordings of acutely dissociated neurons were performed at room temperature using an EPC‐10 amplifier and Patch‐Master software (HEKA, Freiburg). The cells were held at 0 pA under current‐clamp recording and the firing threshold of DRG neurons was measured by a series of 100‐ms depolarizing current injection in 5‐pA steps from 0 pA to elicit the first action potential (AP). To further examine the firing properties of neurons, a large depolarizing current (500 ms, 2‐fold rheobase) was delivered to elicit the cell generating sufficient firing. All recordings were performed on medium‐small diameter neurons as described in previous reports.^19^, ^24^ We measured the rest membrane potential (RMP), the inter‐spike interval (ISI), the after‐hyperpolarization (AHP) 80% duration, the AP amplitude, the AHP amplitude, the threshold potential (TP), and the frequency of AP to evaluate the intrinsic electrophysiological properties of cells. Origin software 9.0 (OriginLab Corporation, Northampton, MA) was used for data analysis.

### 2.16 Electrophysiology

Sensory nerve conduction velocity (SNCV) was determined following previously established method,^25^ and the results were reported in meters per second.

### 2.17 Statistical analysis

All data were expressed as the mean ± SEM. Normality tests were formally underwent using D'Agostino‐Pearson omnibus normality test and nonparametric tests were used for the data which failed the test. One‐way ANOVA followed by Tukey's multiple comparison test was used for comparisons among groups and two‐way ANOVA followed by Bonferroni's post hoc test was used for comparisons among groups with multiple time points. Unpaired two‐tailed mean values of the two groups were compared using the student's *t*‐test. *p* < 0.05 was used to determine the statistical significance of differences. For the statistical analyses, GraphPad Prism 9 for Windows was used (GraphPad Software, La Jolla, CA).

## 3 RESULTS

### 3.1 Morphology and function of mitochondria were changed and mitophagy was inhibited in PDN rats

**FIGURE 1:**
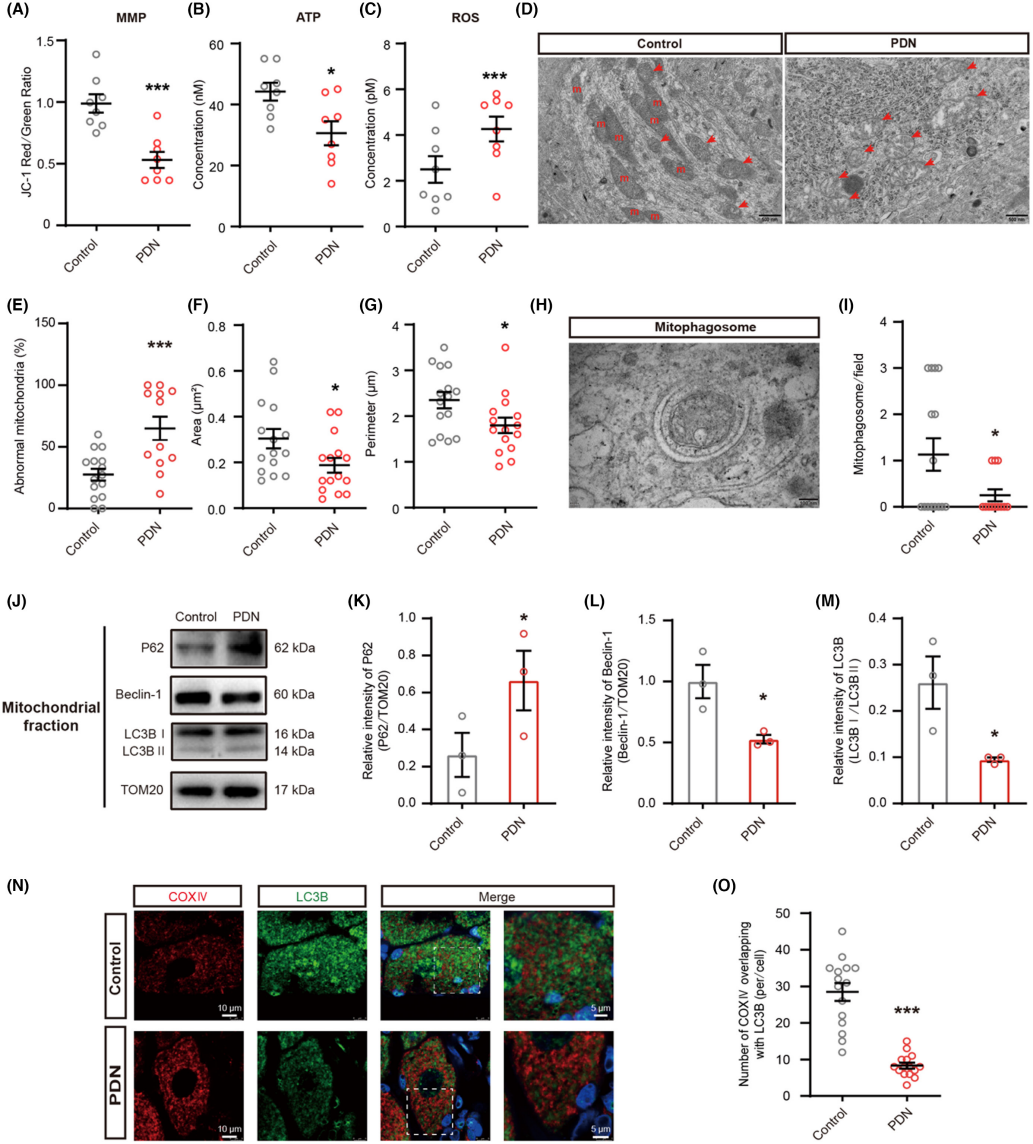
Effects of mitochondria and mitophagy in PDN rats. Effects of MMP, ATP, ROS, mitochondria morphology, the area of mitochondria, the perimeter of mitochondria, and the expression of P62, Beclin‐1, and LC3B in PDN rats. (A) MMP levels of mitochondria in the DRG of rats (***p < 0.001, *n* = 8). (B) ATP levels of mitochondria in the DRG of rats (*p < 0.05, *n* = 8). (C) ROS levels of mitochondria in the DRG of rats (***p < 0.001, *n* = 8). (D–I) TEM examination of mitochondria and mitophagosomes in DRG (*p < 0.05, ***p < 0.001, *n* = 3). (D) Representative electron micrographs of mitochondria structures (red arrows indicate abnormal mitochondria; m: mitochondria; scale bar: 500 nm). (E) The ratio of abnormal mitochondria counts to mitochondrial counts. (F) The area of mitochondria. (G) The perimeter of mitochondria. (H) Representative electron micrographs of mitophagosome structures; scale bar: 100 nm. (I) The number of mitophagosome structures. (J–M) Western blot analysis of P62, Beclin‐1, and LC3B expression in mitochondrial extracts (*p < 0.05, *n* = 3). (J) Representative immunoblots. (K) Quantification for the ratio of P62 to TOM20. (L) Quantification for the ratio of Beclin‐1 to TOM20. (M) Quantification for the ratio of LC3II to LC3I. (N) Co‐localization analysis of confocal laser scanning microscopy images of COX IV (red) and LC3B (green) staining (Scale bar = 5 μm). (O) The degree of co‐localization between COX IV and LC3B (***p < 0.001, *n* = 3). Data are presented as mean ± SEM. *p < 0.05, **p < 0.01, ***p < 0.001, unpaired *t* test for (A–C), (E–G), (I), (K–M), and (O).

The PWT exhibited a decline in PDN rats at day 21 following STZ injection (Figure S1E). Given the importance of mitochondria,^26^ we explored the changes in the quantity and quality of mitochondria in the DRG. These neurons served as nociceptors and were sourced from rats at day 21 after STZ injection. First, we looked at the mitochondrial function. In PDN rats, there was a decrease in MMP and ATP levels, coupled with elevated ROS production, indicating mitochondrial malfunction (Figure 1A–C). The primary characteristic of neuropathy in T2DM is predominantly defined by the deterioration of small nerve fibers.^27^ Then, to assess the potential adaptability of mitochondria in DRG neurons, we conducted an analysis of mitochondrial morphology in the cell bodies of medium‐small‐sized neurons. There was an absence of mitochondrial cristae (indicative of abnormal mitochondria) along with reductions in both mitochondrial perimeter and area (Figure 1D) in the PDN group. Quantitative investigation revealed that rats in the PDN group had considerably more damaged mitochondria and decreased area and perimeter in their DRG neurons (Figure 1E–G).

The buildup of damaged and dysfunctional mitochondria in PDN rats could point to a potential impairment in mitochondrial elimination. Since mitophagy plays a fundamental role in the cellular process of mitochondrial homeostasis,^28^ we observed the changes in mitophagy by TEM (Figure 1H). A decrease in the quantity of mitophagosomes was observed in the PDN group (Figure 1I). To assess the changes in mitophagy, we extracted mitochondrial proteins for immunoblot analysis of autophagy biomarkers P62, Beclin‐1, and LC3B. The expression of P62 was significantly increased in PDN rats, whereas Beclin‐1 and the ratio of LC3II to LC3I were significantly decreased (Figure 1J–M). Mitophagy was also evaluated via co‐staining mitochondrial marker COX IV with the autophagosome marker LC3B (Figure 1N). There was less colocalization of COX IV with LC3B in PDN rats in medium–small‐sized neurons (Figure 1O). These findings supported that mitophagy was inhibited in DRG neurons of PDN rats.

In sum, this in vivo investigation has illuminated that both the structure and function of mitochondria were impaired and the accumulation of these damaged mitochondria could be due to defective mitophagy in PDN rats.

### 3.2 SIRT3 was mainly localized in medium–small size neurons and the expression of SIRT3 was inhibited in PDN rats

**FIGURE 2:**
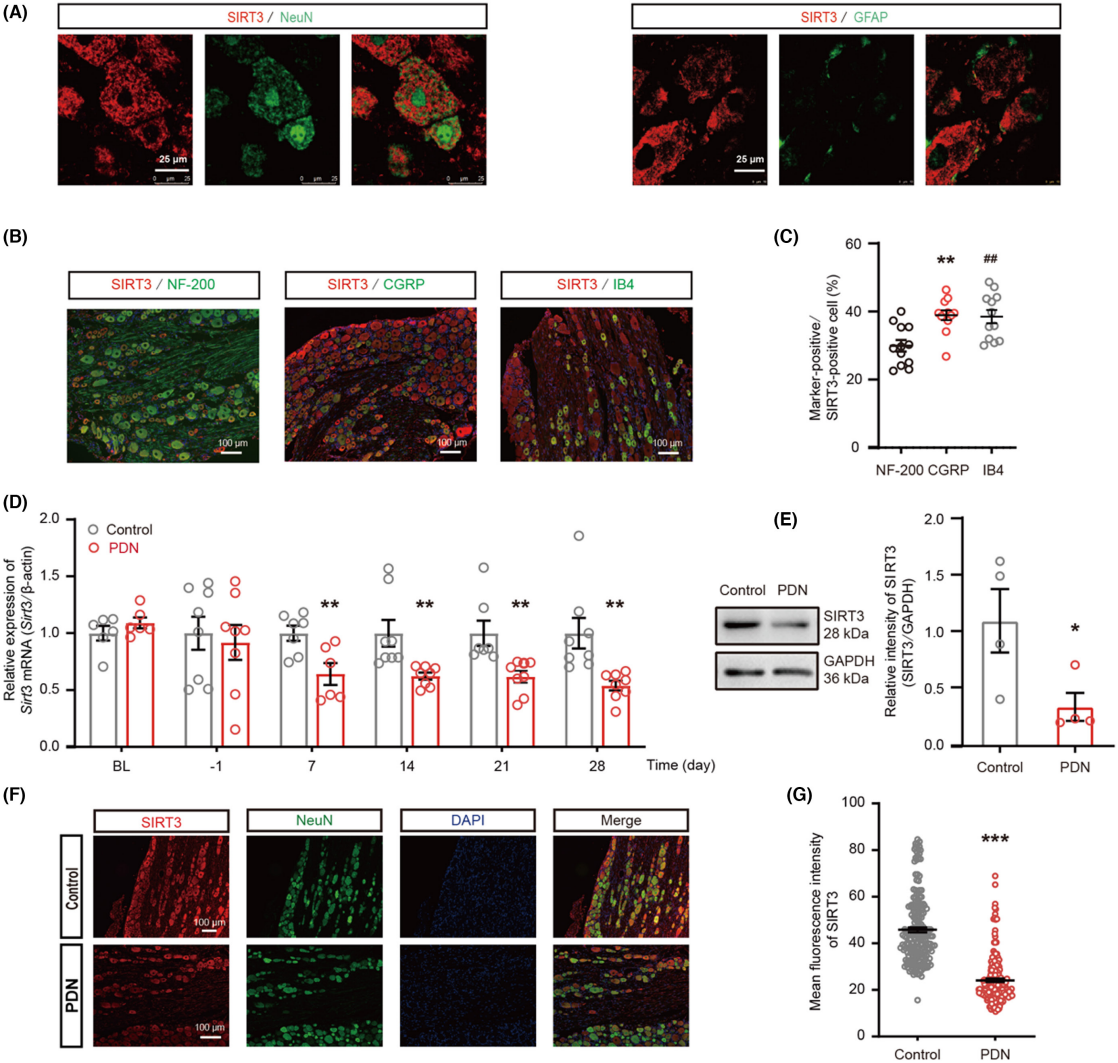
The localization and expression of SIRT3 in PDN rats. (A–C) Distribution of SIRT3 in the DRG on days 21 after STZ injection. (A) SIRT3 was colocalized mostly with neurons (NeuN), and a minority with astrocytes (GFAP) in the DRG (*n* = 3, Scale bar = 25 μm). (B) Representative images of immunofluorescence staining with SIRT3 and large‐diameter NF‐200^+^ neurons, peptide‐rich, medium‐diameter CGRP^+^ neurons, peptide‐poor, small‐diameter isolectin B4 (IB4)‐positive in DRG neurons in PDN rats (*n* = 3, Scale bar = 100 μm). (C) The percentage of NF‐200^+^, CGRP^+^, and IB4^+^ (green) neurons relative to SIRT3 (red) positive cells (**p < 0.01, NF‐200 vs. CGRP; ^##^ *p* < 0.01, NF‐200 vs. IB4). (D) mRNA expression levels of SIRT3 detected by qRT‐PCR in DRG. The BL indicates the baseline, and the numbers below the x‐axis indicate the time after STZ injection (**p < 0.01, *n* = 6–8). (E) The time‐course of SIRT3 protein in the DRG of PDN rats on days 21 after STZ injection (*p < 0.05, *n* = 4). (F) Representative images of immunofluorescence staining with SIRT3 and NeuN in DRG neurons in Control and PDN rats (scale bar = 100 μm). (G) The mean fluorescence intensity of SIRT3 immunostaining in DRG neurons (***p < 0.001, *n* = 3). Data are presented as mean ± SEM. *p < 0.05, **p < 0.01, ***p < 0.001, ^##^ *p* < 0.01, one‐way ANOVA with Tukey's post hoc test for (C); unpaired *t* test for (D), (E), and (G).

SIRT3, a mitochondrial deacetylase, plays a pivotal role in regulating mitochondrial function and protecting neurodegenerative diseases.^29^ In order to ascertain whether there were alterations in SIRT3 levels in PDN rats, we examined the localization of SIRT3 first. Double immunofluorescent staining with SIRT3 and the markers specific for neurons or astrocytes showed that SIRT3 was mostly localized in neurons (Figure 2A). Additionally, immunofluorescent dual‐labeling assays have revealed a co‐localization of SIRT3 with NF‐200 (large‐diameter neurons), CGRP (medium‐diameter and peptide‐rich neurons), and IB4 (peptide‐poor and small‐diameter) markers. The proportions of NF‐200‐positive neurons, CGRP‐positive neurons, and IB4‐positive neurons were quantitatively evaluated relative to the population of SIRT3‐positive cells. The outcomes demonstrated that SIRT3 primarily localized in medium–small‐sized neurons in PDN rats (Figure 2B,C). The time course of *Sirt3* mRNA expression in the DRG was investigated during the development of PDN. The data showed that *Sirt3* mRNA expression began to decline at day 7 following STZ injection (Figure 2D). Western blot results demonstrated that the SIRT3 protein level was significantly decreased at day 21 following STZ injection (Figure 2E). Double staining with SIRT3 and neuron marker NeuN also confirmed that SIRT3 was decreased in the PDN group (Figure 2F,G).

### 3.3 SIRT3 overexpression in DRG upregulated the expression of SIRT3 and attenuated nerve fiber damage, SNCV, pain, and neuronal hyperexcitability in PDN rats

**FIGURE 3:**
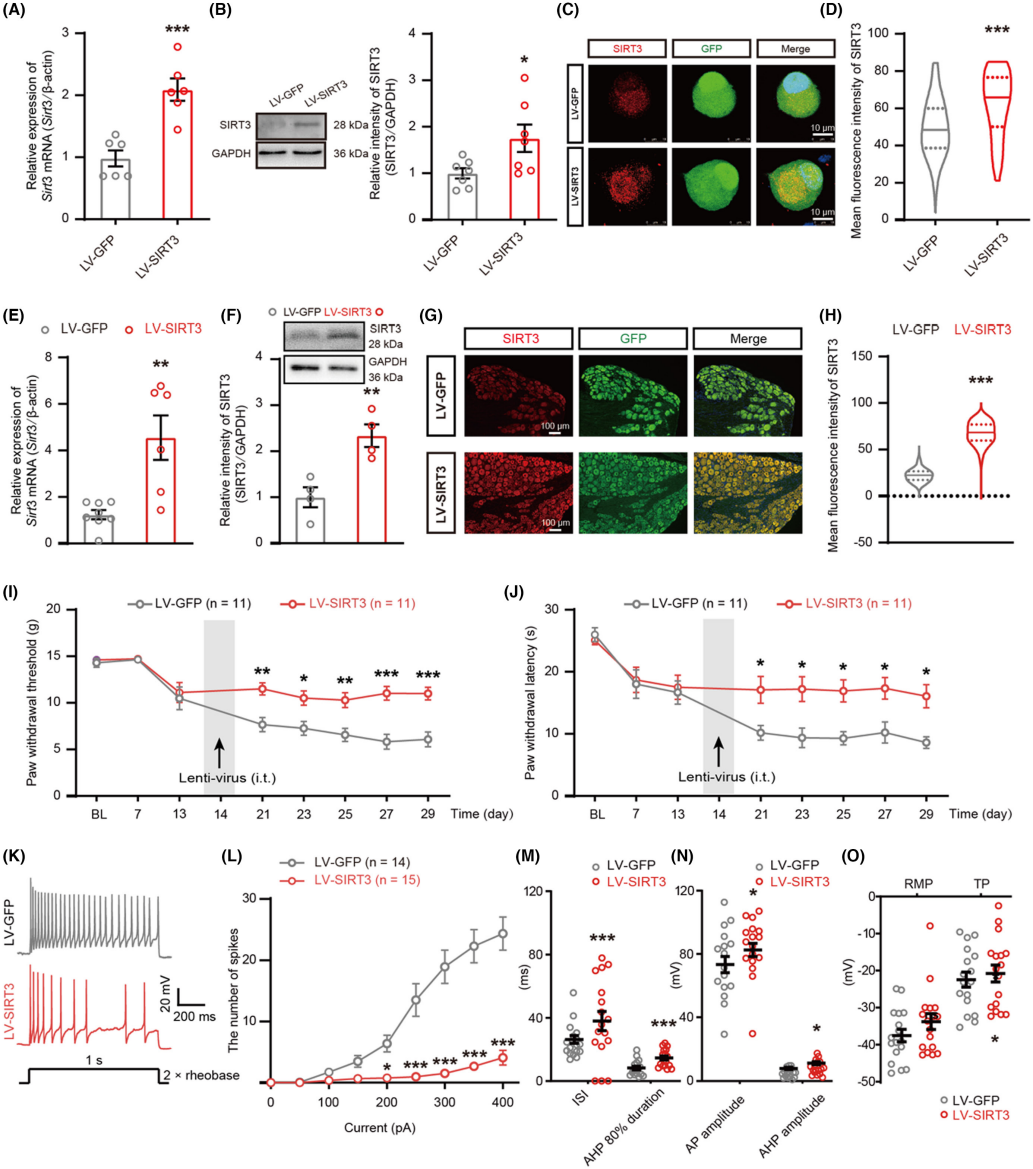
Effects of SIRT3 overexpression on pain hypersensitivity, and DRG neuron hyperexcitability in PDN rats. (A–H) Validation of overexpression viruses on primary cultured DRG neurons (*p < 0.05, ***p < 0.001, *n* = 3–7) and DRG tissues (**p < 0.01, ***p < 0.001, *n* = 3–6). (A and E) qRT‐PCR of SIRT3 overexpression in *Sirt3* mRNA abundance in vitro and in vivo. (B and F) Protein blot of SIRT3 overexpression in vitro and in vivo. (C and G) Immunostaining of SIRT3 with GFP in SIRT3 overexpressing DRG neurons in vitro (scale bar = 10 μm) and in vivo (scale bar = 100 μm). (D and H) Statistical analysis of SIRT3 mean fluorescence intensity in vitro and in vivo. (I and J) PWT and PWL in the hind paws were measured before STZ injection (BL), before LV‐SIRT3 or LV‐GFP injection (day‐13), and at days 7, 14, 21, 23, 25, 27, and 29 after STZ injection (*p < 0.05, **p < 0.01, ***p < 0.001, *n* = 11). (K–O) Electrophysiological analysis of excitability of DRG neurons after LV‐SIRT3 or LV‐GFP injection (*p < 0.05, ***p < 0.001, *n* = 14–17 cells for each group of five rats). (K) Representative traces of neuronal action potentials. (L–O) Statistical analysis of the AP number, ISI, AHP 80% duration, AP amplitude, RMP, and TP in DRG neurons of PDN rats in the LV‐SIRT3 group compared with rats in the LV‐GFP group. Data are presented as mean ± SEM. *p < 0.05, **p < 0.01, ***p < 0.001, unpaired *t* test for (A), (B), (D–F), (H), and (M–O); two‐way ANOVA with Sidak's post hoc test for (I), (J), and (L).

To further explore the relationship between SIRT3 and PDN, we used SIRT3 overexpression lentivirus (LV‐SIRT3) to regulate SIRT3 expression in DRG neurons. The qRT‐PCR, western blot, and immunofluorescent staining results confirmed that SIRT3 expression was upregulated by LV‐SIRT3 in cultured DRG neurons (Figure 3A–D) and PDN rats (Figure 3E–H). To assess the potential influence of SIRT3 on the symptoms of PDN, we examined nerve fibers using TEM (Figure S2A–F). In PDN rats, unmyelinated nerve fibers exhibited a reduced number with the formation of vacuoles (Figure S2A,B) and many abnormal structures (Figure S2C). For the myelinated nerve fibers, larger areas of demyelination and degeneration of axons with full‐thickness demyelination were observed in PDN rats (Figure S2D). In the PDN group, there was a decrease in the number of myelinated nerve fibers per field (Figure S2E) and an increase in the number of damaged axons (Figure S2F). These data signified a notable decrease in axon density and considerable damage to axonal structure in PDN rats. However, these effects were prevented by LV‐SIRT3. Before administration of LV‐SIRT3, rats in the PDN group had a mean SNCV of 27 m/s, whereas those in the LV‐SIRT3 group had a mean SNCV of 48.33 m/s (Figure S2G,H). Furthermore, the overexpression of SIRT3 by LV‐SIRT3 reversed diabetes‐induced hypersensitivity to mechanical and thermal stimulation (Figure 3I,J), and neuronal hyperexcitability in medium–small‐sized neurons, shown as a significant decrease in the number of AP, an increase in the ISI, AHP 80% duration, AP amplitude, AHP amplitude, and TP (Figure 3K–O).

### 3.4 SIRT3 overexpression in DRG restored the morphology and function of mitochondria and rescued the defective mitophagy in PDN rats

**FIGURE 4:**
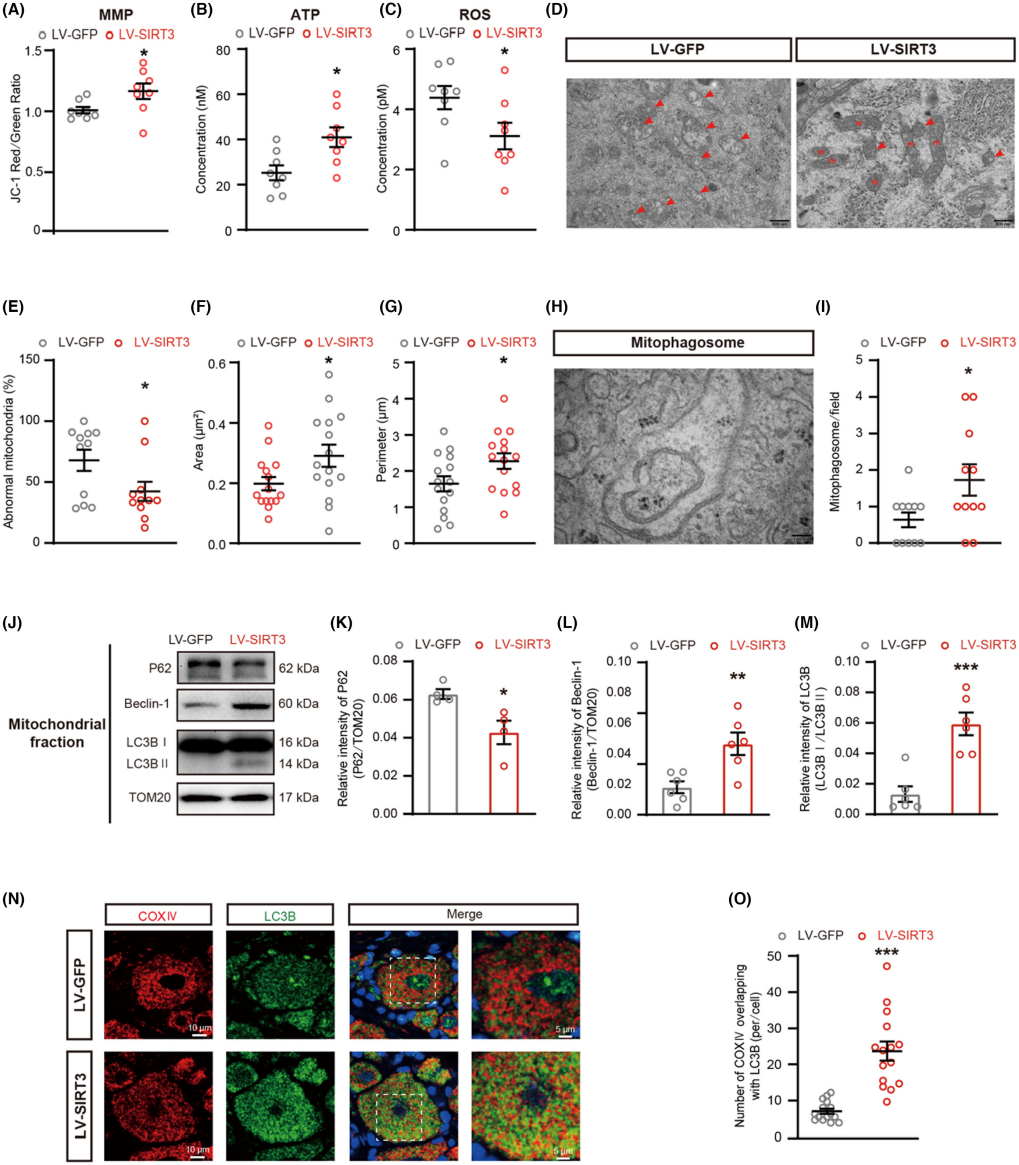
Effects of SIRT3 overexpression on mitochondria and mitophagy in PDN rats. Effects of SIRT3 overexpression on MMP, ATP, ROS, mitochondria morphology, the area of mitochondria, the perimeter of mitochondria, and the expression of P62, Beclin‐1, and LC3B in PDN rats. (A) MMP levels of mitochondria in the DRG of PDN rats treated with LV‐GFP or LV‐SIRT3 (**p < 0.01, *n* = 8). (B) ATP levels of mitochondria in the DRG of rats (*p < 0.05, *n* = 8). (C) ROS levels of mitochondria in the DRG of rats (*p < 0.05, *n* = 8). (D–I) TEM examination of mitochondria and mitophagosomes in PDN rats after LV‐GFP or LV‐SIRT3 injection (*p < 0.05, *n* = 3). (D) Representative electron micrographs of mitochondria structures (red arrows indicate abnormal mitochondria; m: mitochondria; scale bar: 500 nm). (E) The ratio of abnormal mitochondria counts to mitochondrial counts. (F) The area of mitochondria. (G) The perimeter of mitochondria. (H) Representative electron micrographs of mitophagosome structures; scale bar: 100 nm. (I) The number of mitophagosome structures. (J–M) Western blot analysis of P62, Beclin‐1, and LC3B expression in mitochondrial extracts (*p < 0.05, **p < 0.01, ***p < 0.001, *n* = 4–6). (J) Representative immunoblots. (K) Quantification for the ratio of P62 to TOM20. (L) Quantification for the ratio of Beclin‐1 to TOM20. (M) Quantification for the ratio of LC3II to LC3I. (N) Co‐localization analysis of confocal laser scanning microscopy images of COX IV (red) and LC3B (green) staining. (O) the degree of co‐localization between COX IV and LC3B (***p < 0.001, *n* = 3). Data are presented as mean ± SEM. *p < 0.05, **p < 0.01, ***p < 0.001, unpaired *t* test for (A–C), (E–G), (I), (K–M), and (O).

The overexpression of SIRT3 through LV‐SIRT3 restored the MMP and ATP levels and reduced the production of ROS (Figure 4A–C) and the number of damaged mitochondria in PDN rats (Figure 4D–G). There were more mitophagosome structures in the cell bodies of medium‐small size neurons after LV‐SIRT3 injection (Figure 4H,I). SIRT3 overexpression reversed the increase of P62 and reversed the reduction of Beclin‐1 and the ratio of LC3II to LC3I (Figure 4J–M). The overexpression of SIRT3 resulted in increased colocalization of COX IV with LC3B in medium–small‐sized neurons (Figure 4N,O). These results implied that the inhibited mitophagy in the DRG of PDN rats was reversed by LV‐SIRT3 injection.

### 3.5 FoxO3a, PINK1, and Parkin were mainly localized in DRG neurons, and their expression in PDN rats was restored by LV‐SIRT3

**FIGURE 5:**
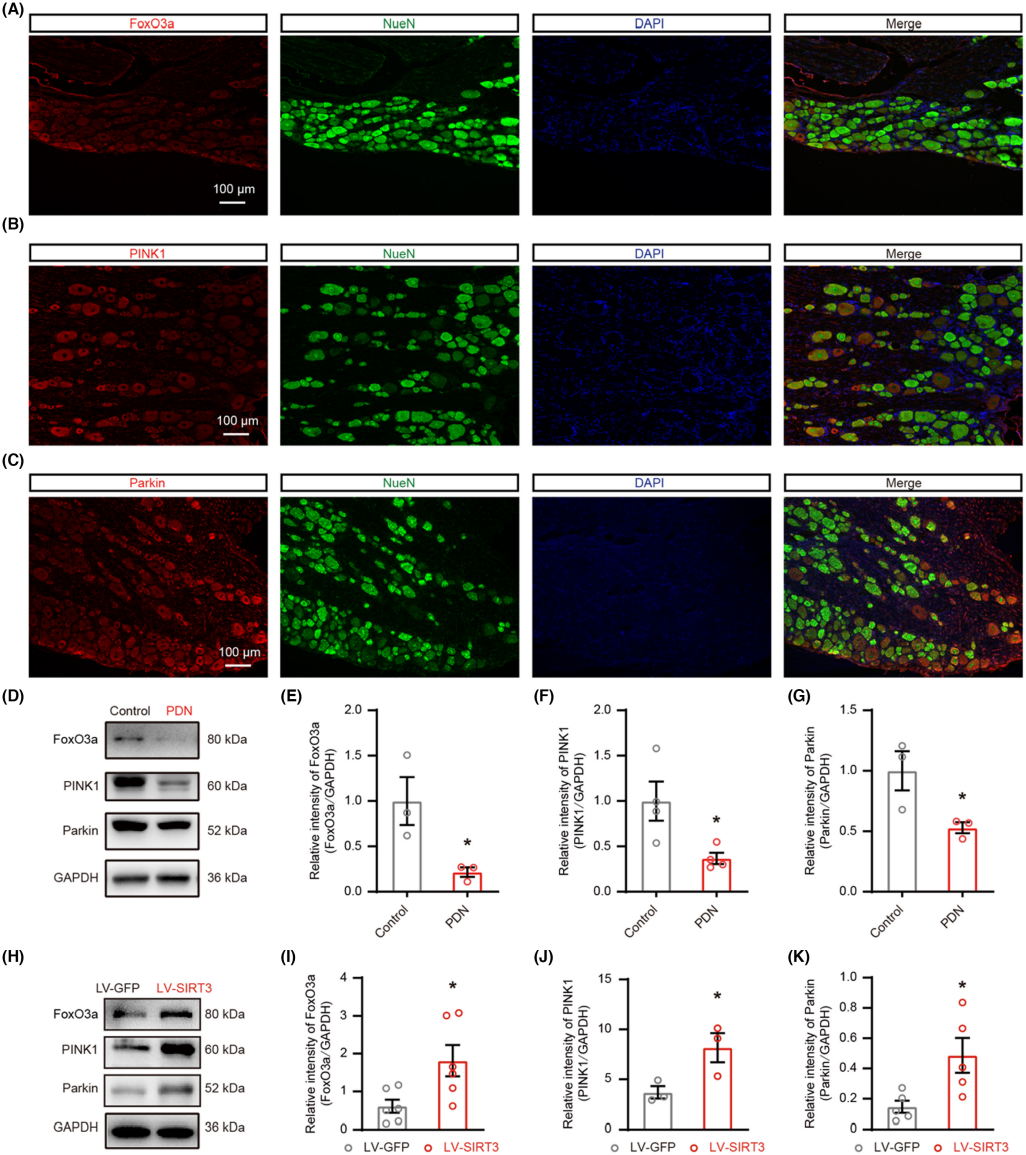
The localization and expression of FoxO3a, PINK1, and Parkin. The localization and expression of FoxO3a, PINK1, and Parkin and impacts of SIRT3 overexpression on the expression FoxO3a, PINK1, and Parkin in PDN rats. (A) Representative images of immunofluorescence staining with FoxO3a and NeuN in DRG neurons in PDN rats. (B) Representative images of immunofluorescence staining with PINK1 and NeuN in DRG neurons in PDN rats. (C) Representative images of immunofluorescence staining with Parkin and NeuN in DRG neurons in PDN rats. (D–K) Protein blot analysis of FoxO3a, PINK1, and Parkin in whole‐cell extracts was performed in the DRG of rats. In H‐K, PDN rats were injected with LV‐SIRT3 or LV‐GFP (*p < 0.05, *n* = 3–6). (D) The representative blots graph. (E–G) Statistical analysis showing the relative intensities of FoxO3a, PINK1, and Parkin. (H) The representative blots graph. (I–K) Statistical analysis of the relative intensities of FoxO3a, PINK1, and Parkin. Data are presented as mean ± SEM. *p < 0.05, **p < 0.01, ***p < 0.001, unpaired *t* test for (E–G) and (I–K).

Since FoxO3a is a direct target of SIRT3,^9^, ^11^ and the PINK1‐Parkin pathway is a well‐known mitophagy pathway,^30^ we explored the presence of FoxO3a, PINK1, and Parkin in DRG neurons. Double immunofluorescent staining revealed overlapping staining of FoxO3a, PINK1, and Parkin with NeuN in DRG in PDN rats (Figure 5A–C). We measured the protein levels of FoxO3a, PINK1, and Parkin in DRG neurons, and found that the expression of FoxO3a, PINK1, and Parkin was decreased in the PDN group (Figure 5D–G). Overexpression of SIRT3 by LV‐SIRT3 enhanced the expression of FoxO3a, PINK1, and Parkin as demonstrated by western blot (Figure 5H–K). Taken together, these results demonstrated that SIRT3 acted upstream to FoxO3a, PINK1‐Parkin mitophagy pathway, which may play a potential role in PDN.

### 3.6 Knockdown of FoxO3a in DRG aggravated nerve fiber damage, increased neuronal hyperexcitability and pain hypersensitivity, changed the morphology and function of mitochondria, inhibited the activation of mitophagy, and the expression of FoxO3a, PINK1, and Parkin in overexpressing SIRT3 rats with PDN

**FIGURE 6:**
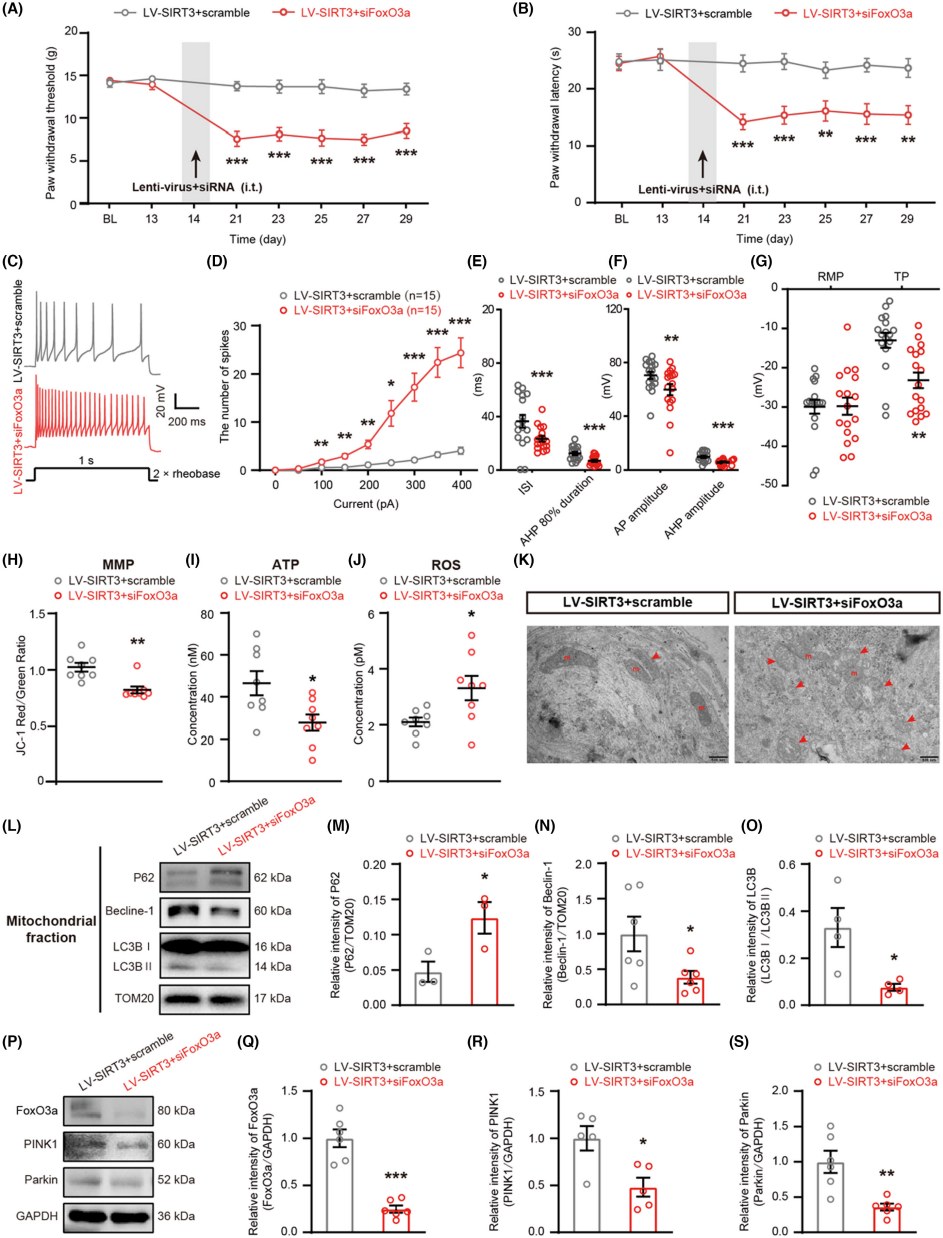
FoxO3a knockdown counteracted the effect of SIRT3 overexpression. (A and B) PWT and PWL in the hind paws were measured before STZ injection (BL), before LV‐SIRT3+scramble or LV‐SIRT3+siFoxO3a injection (day−13), and at days 7, 14, 21, 23, 25, 27, and 29 after STZ injection (**p < 0.01, ***p < 0.001, *n* = 11). (C–G) Electrophysiological analysis of DRG neurons after LV‐SIRT3+scramble or LV‐SIRT3+siFoxO3a injection (*p < 0.05, **p < 0.001, ***p < 0.001, *n* = 16–17 cells for each group of five rats). (C) Representative traces of neuronal AP. (D–G) Statistical analysis of the AP number, ISI, AHP 80% duration, AP amplitude, RMP, and TP in DRG neurons of PDN rats. (H) MMP levels of mitochondria in the DRG of rats (**p < 0.01, *n* = 8). (I) ATP levels of mitochondria in the DRG of rats (*p < 0.05, *n* = 8). (J) ROS levels of mitochondria in the DRG of rats (*p < 0.05, *n* = 8). (K) Representative electron micrographs of mitochondria structures (red arrows indicate abnormal mitochondria; m: mitochondria; scale bar: 500 nm). (L–O) Western blot analysis of P62, Beclin‐1, and LC3B expression in mitochondrial extracts (*p < 0.05, *n* = 3–6). (L) Representative immunoblots. (M) Quantification for the ratio of P62 to TOM20. (N) Quantification for the ratio of Beclin‐1 to TOM20. (O) Quantification for the ratio of LC3II to LC3I. (P) Representative blots of FoxO3a, PINK1, and Parkin in whole‐cell extracts after LV‐SIRT3+scramble or LV‐SIRT3+siFoxO3a injection. (Q–S) Statistical analysis of the relative intensities of FoxO3a, PINK1, and Parkin (*p < 0.05, **p < 0.01, ***p < 0.001, *n* = 5–6). Data are presented as mean ± SEM. *p < 0.05, **p < 0.01, ***p < 0.001, two‐way ANOVA with Sidak's post hoc test for (A), (B), and (D); unpaired *t* test for (E–J), (M–O), and (Q–S).

To confirm the contribution of FoxO3a, LV‐SIRT3 and FoxO3a siRNA were intrathecally injected to knock down FoxO3a while maintaining SIRT3 overexpression in PDN rats. Knockdown of FoxO3a attenuated the analgesic effect of LV‐SIRT3 (Figure 6A–G) and mitigated its inhibitory effect on damaged nerve fibers (Figure S3A–H). FoxO3a knockdown also erased the effect of SIRT3 overexpression on mitochondria (Figure 6H–K and Figure S3I–K) and mitophagy (Figure 6L–O and Figure S3L–O). Downregulation of FoxO3a led to a decrease in the expression of PINK1 and Parkin, suggesting that PINK1‐Parkin was regulated by FoxO3a (Figure 6P–S). Thus, these data indicated that FoxO3a was crucial for SIRT3‐dependent mitophagy.

## 4 DISCUSSION

In this study, we found that damaged mitochondria were accumulated and mitophagy was inhibited in cell bodies of the medium–small‐sized DRG neurons in PDN rats; SIRT3 primarily localized in medium–small‐sized neurons and the expression of SIRT3 in PDN rats was reduced; the hyperalgesia, damaged nerve fiber and mitochondria in PDN rats were attenuated by treatment with LV‐SIRT3 through activating the SIRT3‐mediated FoxO3a‐PINK1‐Parkin mitophagy pathway; FoxO3a was crucial for SIRT3‐dependent mitophagy. Altogether, our results supported that mitophagy dysfunction might be an important cause of PDN and SIRT3 might be a novel therapeutic target for PDN treatment.

Defects in mitochondrial morphology and dynamics could impair mitochondrial function, affecting neuronal survival and function.^3^ In our study, DRG neurons in PDN rats had fragmented and vacuolated mitochondria. Similar morphological changes in mitochondria were observed in DRG in type 1 diabetes mellitus (T1DM) rats.^31^ Since MMP is a driving force for the transport of ions and proteins which are necessary for healthy mitochondria, mitochondrial function can be assessed by monitoring MMP changes.^32^ In ATP generation, mitochondria rely on the stability of the MMP. A decrease in MMP could lead to mitochondrial matrix swelling and impair OXPHOS function, thereby disrupting the normal synthesis of ATP.^33^ Additionally, we quantified the DCFH‐DA signal to assess cellular levels of ROS, which are an inevitable consequence of impaired mitochondrial function.^34^ Our data demonstrated that DRG neurons' MMP and ATP levels in PDN rats with T2DM were reduced, and the ROS production was increased.

The balance of fission, fusion, and autophagy determines the morphology and dynamics of mitochondria.^35^ Dysfunctional mitochondria are required to be eliminated by mitophagy on time. Mitophagy has been found to be altered in various diabetic complications.^36^ There is evidence that diabetic retina,^37^ diabetic cardiomyopathy,^11^ and diabetic nephropathy^38^ are associated with an impairment of mitophagy. The studies on the role of mitophagy in PDN are limited and the results are controversial. Defective mitophagy was found in DRG neurons in db/db mice,^39^ but activated mitophagy in spinal cords^40^ and DRG^41^ was reported in the T1DM mice model. In our study, combined HFD and low‐dose STZ rat models were chosen since it is analogous to the development of human T2DM,^42^ and it is the most frequently used in the studies of PDN.^43^ Our study unveiled that mitophagy was inhibited in medium–small‐sized DRG neurons, as evidenced by TEM revealing a decrease in mitophagosome formation and the analysis of altered autophagy biomarkers in extracted mitochondrial protein. The increase of mitophagy in T1DM mice in a previous study may reflect an adaptive response to eliminate defective mitochondria at a single time point; however, it is likely that mitophagy would eventually decrease.^36^ In our study, using a HFD and low‐dose STZ model, reduced mitophagy capability coincided with notable mitochondrial fragmentation and dysfunction, correlating with PDN.

The SIRT family is a group of highly conserved NAD^+^‐dependent protein deacetylases with seven subtypes in mammals, namely SIRT1 through SIRT7.^44^ Each subtype of SIRT exhibits distinct cellular localization patterns. SIRT2 primarily localizes to the cytoplasm, while SIRT1, SIRT6, and SIRT7 are predominantly nuclear. In contrast, SIRT3, SIRT4, and SIRT5 are chiefly found in the mitochondria.^45^ Given our observation of mitochondrial alterations in PDN rats and the lack of correlation between SIRT4 and SIRT5 with DRG or neuropathy, we have chosen to focus our investigation on SIRT3. In the neuropathic pain and inflammatory pain model, SIRT3 depletion induces mitochondrial antioxidant enzyme dysfunction and thus increased oxidative stress.^46^, ^47^ SIRT3 is primarily localized to the inner mitochondrial membrane and recent efforts have shed light on the potential role of SIRT3 in mitophagy.^13^, ^48^ A previous study revealed that T1DM prompts cardiac dysfunction, accompanied by suppressed mitophagy, which is exacerbated by SIRT3 knockdown.^11^ In our current study, SIRT3 was proved to be primarily localized in medium–small‐sized DRG neurons. A previous study focused on spinal dorsal horn also indicated SIRT3 is mainly present on neurons rather than microglial cells.^9^ A reduction in the expression of SIRT3 was found in PND rats. Through overexpression of SIRT3 using LV‐SIRT3, we observed an increase in mitophagosome structures and a reversal of the inhibited mitophagy. The latter was supported by changes in mitochondrial P62 and Beclin‐1 levels, as well as alterations in the ratio of LC3II to LC3I. Although our study did not directly investigate the effects of SIRT3 on the spinal cord, we acknowledge the importance of spinal cord involvement in pain modulation. Both LV‐SIRT3 and its control, LV‐GFP, administered intrathecally have the potential to directly influence spinal cord neurons. As the spinal cord is integral in processing and transmitting sensory signals to the brain, the presence of SIRT3 on spinal cord neurons has been well‐documented in previous studies.^9^, ^47^ These findings underscored the importance of considering spinal cord involvement in our investigation. Moreover, given that our study primarily focused on elucidating the mechanism of SIRT3 action within DRG in PDN, we acknowledge the intricate interplay between the DRG and the spinal cord in nociception. The DRG houses cell bodies of sensory neurons responsible for transmitting signals from peripheral tissues to the spinal cord. Our research confirmed the significant role of modulating SIRT3 within the DRG in nociceptive pain perception and sensory transmission. In light of these considerations, while our study did not directly address the effects of SIRT3 on the spinal cord, we recognize its potential implications and emphasize the importance of future investigations to comprehensively elucidate the involvement of SIRT3 in pain modulation across different neural structures.

FoxO3a is a member of the FOXO subfamily of forkhead transcription factors.^14^ Previous studies have provided evidence that SIRT3‐mediated deacetylation of FoxO3a further regulates FoxO3a phosphorylation, ubiquitination, and degradation, thereby stabilizing FoxO3a proteins.^16^, ^49^, ^50^ Our data showed that the levels of SIRT3 and FoxO3a were decreased in PDN and overexpression of SIRT3 increased the expression of FoxO3a. Knocking down FoxO3a while maintaining SIRT3 overexpression counteracted the analgesic effect of LV‐SIRT3. Our findings were consistent with a previous study indicating the involvement of SIRT3 in regulating the expression of FoxO3a in PDN model.^9^ The regulation of FoxO3a by SIRT3 primarily occurs indirectly, whereby the deacetylation of FoxO3a by SIRT3 subsequently influences its phosphorylation and ubiquitination.^9^

Currently, PINK1‐Parkin‐mediated mitophagy is the most widely described mitophagy pathway in mammals.^51^, ^52^ PINK1, a serine/threonine kinase, plays a vital role in detecting damaged mitochondria within the cell. Upon sensing mitochondrial damage, PINK1 initiates a signaling cascade that involves the activation of Parkin. Subsequently, PINK1 and Parkin work together to degrade the damaged mitochondria through mitophagy.^53^ In order to examine the involvement of FoxO3a in PINK1‐Parkin‐mediated mitophagy, we initially employed the immunofluorescence staining technique. Our findings revealed the colocalization of FoxO3a, PINK1, and Parkin within the neurons of DRG in PDN rats. The expression levels of PINK1, Parkin, and mitophagy were significantly elevated upon overexpression of SIRT3. Conversely, blocking FoxO3a in SIRT3‐overexpressing rats resulted in a downregulation of PINK1, Parkin, and the level of mitophagy. There are several studies analyzing the changes of the FoxO3a‐PINK1‐Parkin pathway in different DM models. Similar to our study, in retinal pigment epithelium under a high glucose environment, the FoxO3a/PINK1‐Parkin pathway is inhibited, intracellular ROS level is increased, and mitophagy is attenuated.^54^ Peritoneal macrophage from the T2DM mice also shows significantly decreased PINK1 expression. Acetylation of FoxO3a induced by palmitate results in decreased PINK1‐mediated mitophagy and increased activation of the NLRP3 inflammasome.^55^ However, in a 3D organoid ex vivo model of pancreatic β cells, after diabetic stress, Parkin is recruited to the mitochondria, Parkin‐mediated mitophagy is induced, and FoxO3a causes a negative feedback loop by inhibiting the Plk3‐PINK1‐Parkin axis for mitophagy.^56^ The variations in research findings among different studies could be attributed to differences in cell types, in vivo or in vitro experimental models, and the stage of diabetes being studied (early or late). Extensive research is necessary to investigate the mechanisms responsible for the dysregulation of mitophagy in various diabetic complications.

In summary, damaged mitochondria were accumulated and mitophagy was inhibited in medium–small‐sized DRG neurons in PDN rats. Overexpression of SIRT3 restored the inhibited mitophagy by activating the FoxO3a‐PINK1‐Parkin signaling pathway. Our findings indicate that SIRT3 and FoxO3a play a critical role in facilitating PINK1‐Parkin mitophagy. This study offers a novel perspective on understanding the pathogenesis of PDN and identifies a potential therapeutic target for future development.

## FUNDING INFORMATION

This work was supported by the National Natural Science Foundation of China, nos. 82001329, 82071411, 82371227, and 82171226; Beijing Municipal Natural Science Foundation, nos. 7204325 and 7222105; and the Key Research Foundation from Peking University Third Hospital, no. BYSYZD2019036.

## CONFLICT OF INTEREST STATEMENT

The authors declare that they have no conflict of interest.

## Supporting information


Figure S1.

Figure S2.

Figure S3.

## Data Availability

The key data are contained in the figures and additional files. The datasets used and/or analyzed during this study can be further obtained from the corresponding author on reasonable request.
