# Supplementary material for: SIRT3 alleviates painful diabetic neuropathy by mediating the FoxO3a‐PINK1‐Parkin signaling pathway to activate mitophagy
Source: CNS Neurosci Ther. 2024 Apr 4;30(4):e14703. doi: 10.1111/cns.14703 (PMC10993345; doi:10.1111/cns.14703)
Supplement: Supplementary file 1 — Figure S1. [file CNS-30-e14703-s002.docx]

Figure S1


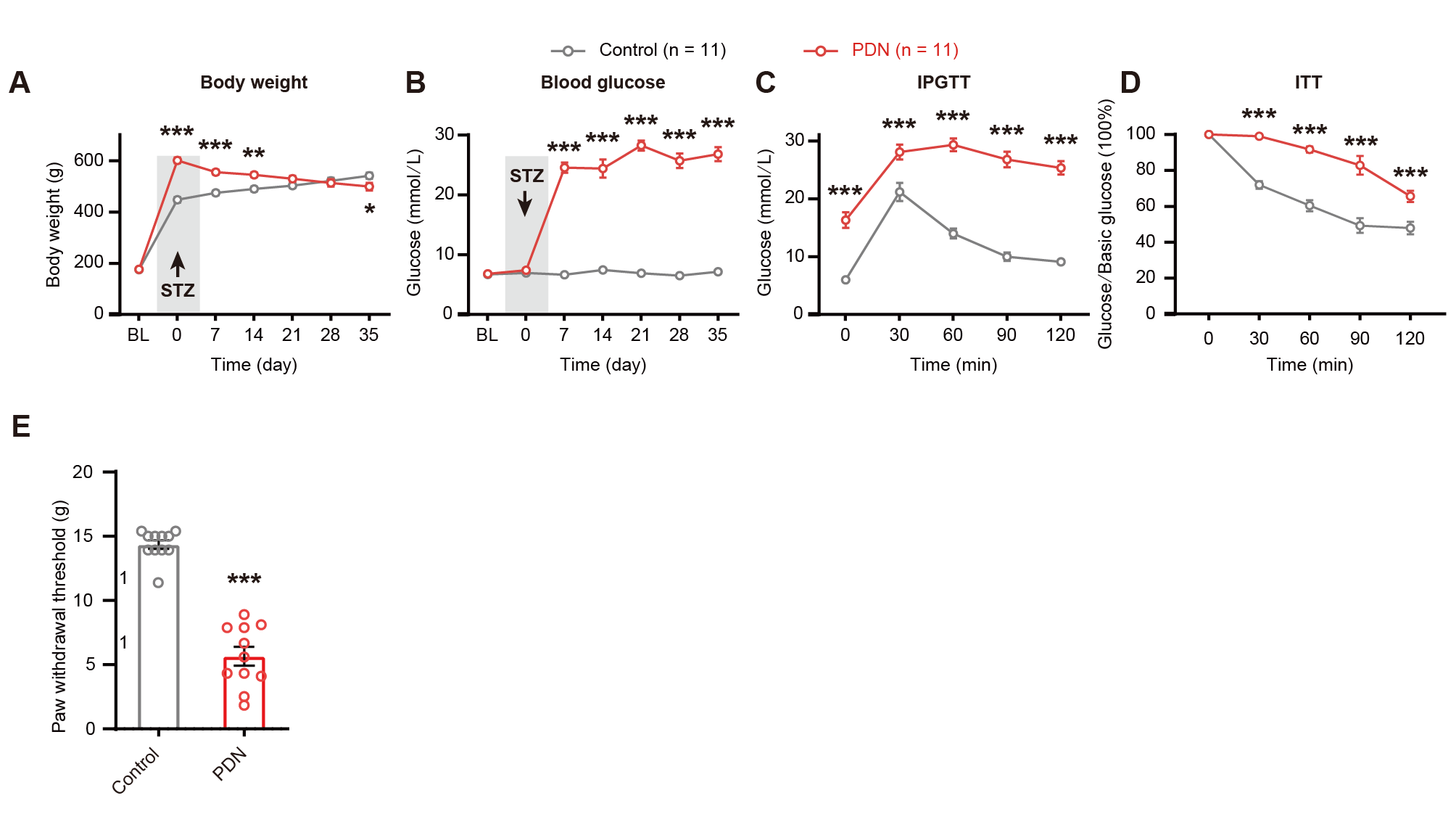


Figure S1: The model of high-fat diet (HFD)/low-dose streptozotocin (STZ) induced rats.

(A) (B) Time course of body weight and blood glucose in rats in the PDN group (n = 11) compared to rats in the Control group (***p* < 0.01, ****p*<0.001, n = 11). (C) Intraperitoneal glucose tolerance test (IPGTT) of rats on days 7 after STZ injection (****p*<0.001, n = 11). (D) Insulin tolerance test (ITT) of rats on days 14 after STZ injection (****p*<0.001, n = 11). (E) Paw withdrawal threshold (PWT) of rats on days 21 after STZ injection (****p*<0.001, n = 11). Data are presented as mean ± SEM. **p* <0.05, ***p* < 0.01, ****p*<0.001, two-way ANOVA with Sidak’s *post hoc* test for (A-D); unpaired t test for (E).
