# Supplementary material for: SIRT3 alleviates painful diabetic neuropathy by mediating the FoxO3a‐PINK1‐Parkin signaling pathway to activate mitophagy
Source: CNS Neurosci Ther. 2024 Apr 4;30(4):e14703. doi: 10.1111/cns.14703 (PMC10993345; doi:10.1111/cns.14703)
Supplement: Supplementary file 2 — Figure S2. [file CNS-30-e14703-s003.docx]

Figure S2


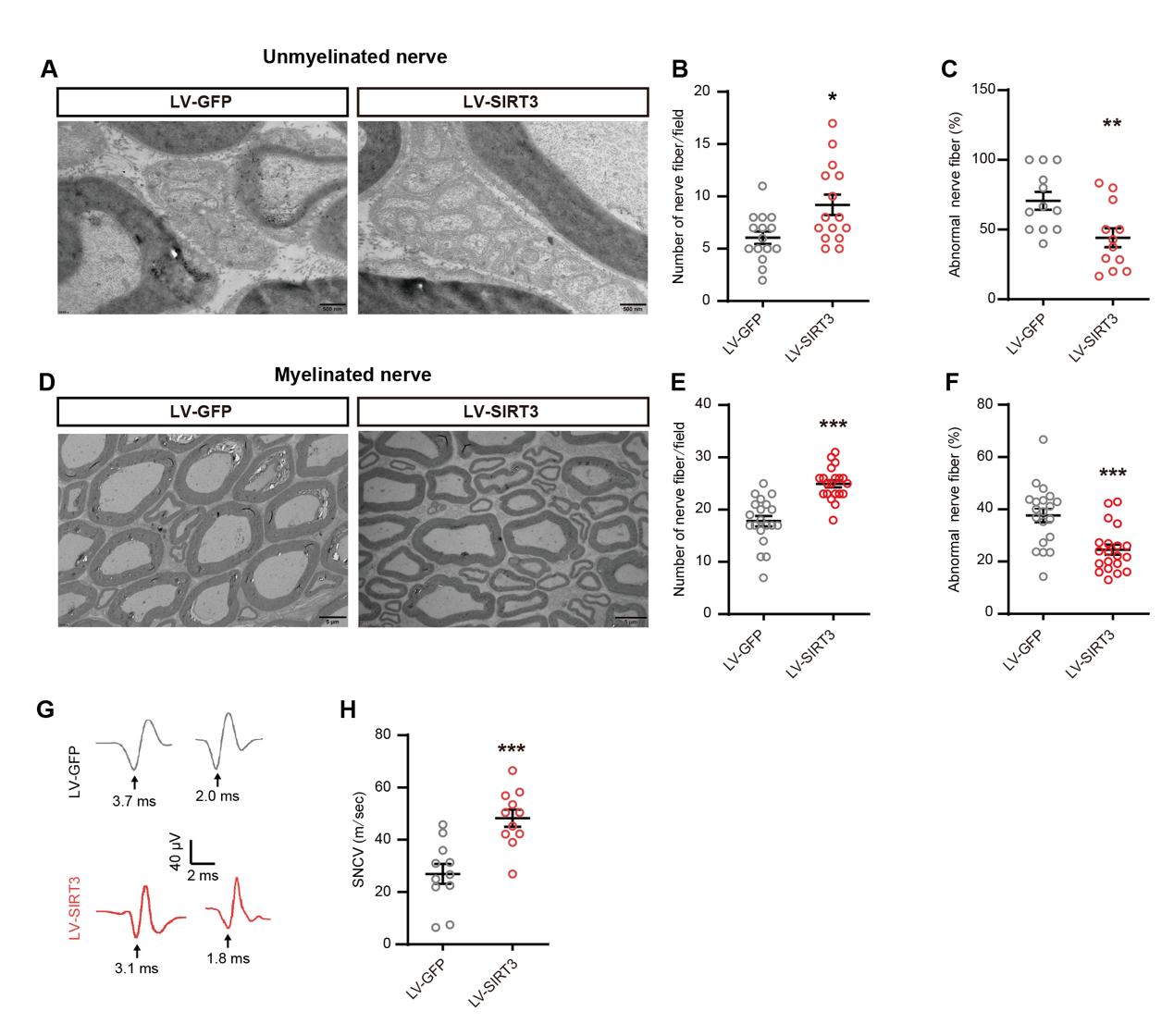


Figure S2: Effects of SIRT3 overexpression on nerve fiber and SNCV in PDN rats.

(A) DRG unmyelinated axons after overexpression of SIRT3. Note the recovery of fiber loss and the resolution of swollen fibers (n = 11-15 fields of view for each group of three rats). (B) Statistical analysis of the number of unmyelinated fibers (**p* < 0.05). (C) Statistical analysis of the proportion of abnormal unmyelinated fibers relative to the total number of unmyelinated fibers (***p* < 0.01). (D) Myelinated axons in the DRG. Note the recovery of myelinated fiber loss and the resolution of myelin splitting (n = 20 fields of view for each group of three rats). (E) Statistical analysis of the number of myelinated fibers (****p* < 0.001). (F) Statistical analysis of the proportion of abnormal myelinated fibers relative to the total number of myelinated fibers (****p* < 0.001). (G) Representative traces of the SNCV in rats. (H) Statistical analysis of the SNCV (****p* < 0.001, n = 11). Data are presented as mean ± SEM. **p* <0.05, ***p* < 0.01, ****p*<0.001, unpaired t test for (B), (C), (E), (F), (H).
