# Supplementary material for: SIRT3 alleviates painful diabetic neuropathy by mediating the FoxO3a‐PINK1‐Parkin signaling pathway to activate mitophagy
Source: CNS Neurosci Ther. 2024 Apr 4;30(4):e14703. doi: 10.1111/cns.14703 (PMC10993345; doi:10.1111/cns.14703)
Supplement: Supplementary file 3 — Figure S3. [file CNS-30-e14703-s001.docx]

**Figure S3**


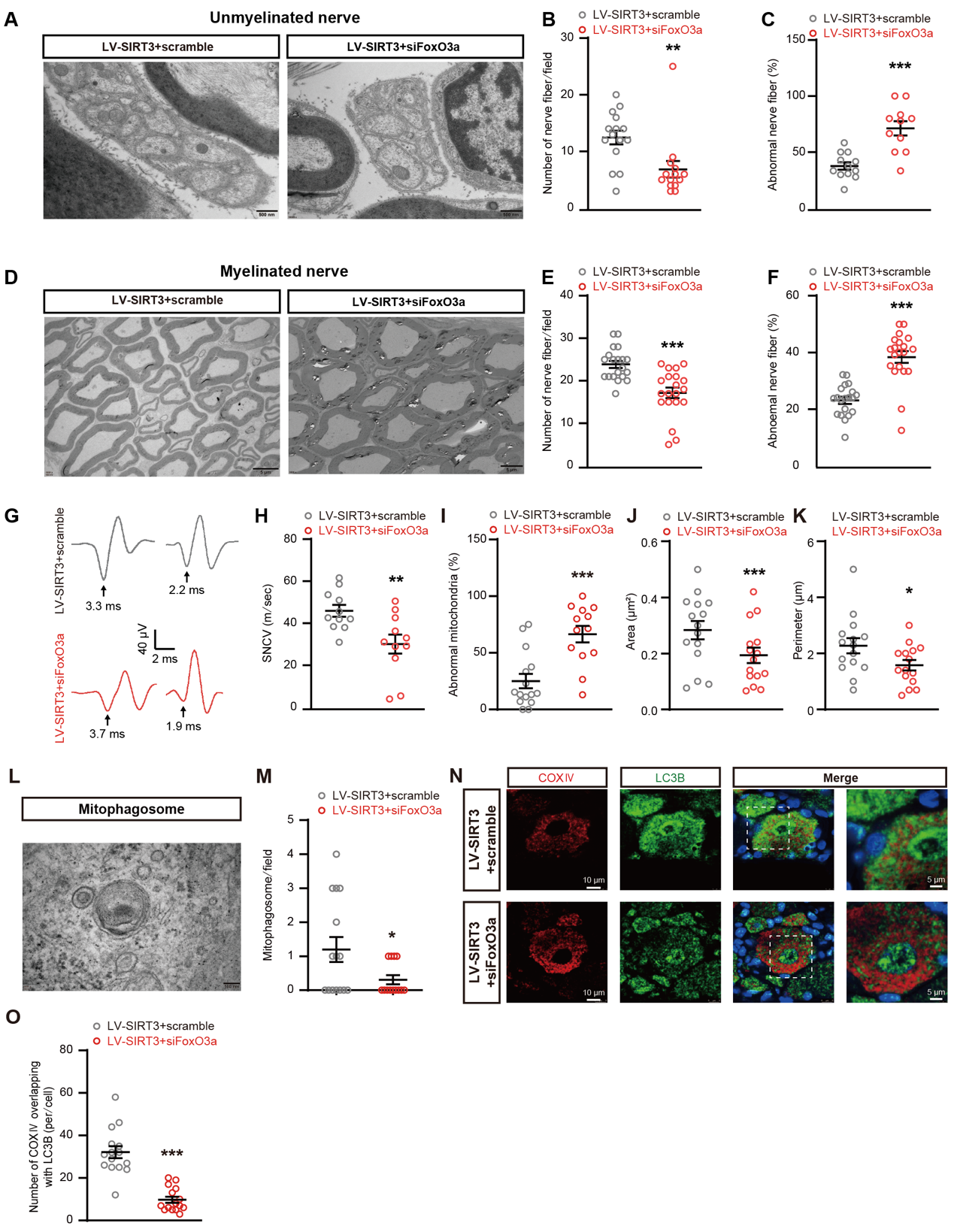


Figure S3: Effects of FoxO3a knockdown in SIRT3-overexpressing rats with PDN.

(A) DRG unmyelinated axons after overexpression of SIRT3 and knockdown of FoxO3a . Note the loss of unmyelinated fibers and the swollen fibers (n = 11-15 fields of view for each group of three rats). (B) Statistical analysis of the number of unmyelinated fibers (***p* < 0.01). (C) Statistical analysis of the proportion of abnormal unmyelinated fibers relative to the total number of unmyelinated fibers (****p* < 0.001). (D) Myelinated axons in the DRG. Note evidence of myelinated fiber loss and occasional fibers with myelin splitting (n = 20 fields of view for each group of three rats). (E) Statistical analysis of the number of myelinated fibers (****p* < 0.001). (F) Statistical analysis of the proportion of abnormal myelinated fibers relative to the total number of myelinated fibers (****p* < 0.001). (G) Representative traces of the SNCV in rats. (H) Statistical analysis of the SNCV (***p* < 0.01, n = 11). (I) The ratio of abnormal mitochondria counts to total mitochondrial counts (****p* < 0.001, n = 3). (J) The area of mitochondria. (K) The perimeter of mitochondria. (L) Representative electron micrographs of mitophagosome structures; scale bar: 100 nm. (M) Quantification for the number of mitophagosome structures (**p* < 0.05, n = 3). (N) Co-localization analysis of confocal laser scanning microscopy images of COX Ⅳ (red) and LC3B (green) staining; scale bar: 10 μm. (O) the degree of co-localization between COX Ⅳ and LC3B (****p* < 0.001, n = 3). Data are presented as mean ± SEM. **p* <0.05, ***p* < 0.01, ****p*<0.001, unpaired t test for (B), (C), (E), (F), (H-K), (M), (O).
